# Diabetes Distress and Unmet Support Needs Hinder Optimal Care for Adolescents With Type 2 Diabetes: A Mixed Methods Study

**DOI:** 10.1155/pedi/5574666

**Published:** 2025-01-17

**Authors:** Dana Spajic, Jacqueline Curran, Yasmin Luu, Mark A. E. Shah, Gitanjali Subramani, Radhika James, Melissa Oxlad, Jane Speight, Alexia S. Peña

**Affiliations:** ^1^Discipline of Paediatrics, Women's and Children's Hospital, The University of Adelaide and Robinson Research Institute, 72 King William Road, North Adelaide 5006, South Australia, Australia; ^2^Endocrinology and Diabetes, Perth Children's Hospital, 15 Hospital Avenue, Nedlands 6009, Western Australia, Australia; ^3^Endocrine and Diabetes Department, Women's and Children's Hospital, 72 King William Road, North Adelaide 5006, South Australia, Australia; ^4^School of Psychology, The University of Adelaide, Hughes North Terrace 5005, Adelaide South Australia, Australia; ^5^School of Psychology, Institute for Health Transformation, Deakin University, 1 Gheringhap Street, Geelong 3220, Victoria, Australia; ^6^The Australian Centre for Behavioural Research in Diabetes, Level 7/14−20 Blackwood Street, North Melbourne, Victoria 3051, Australia

**Keywords:** adolescents, diabetes distress, healthcare needs, type 2 diabetes, youth

## Abstract

**Objectives:** Adolescents with type 2 diabetes (T2D) are more likely than those with type 1 diabetes (T1D) to develop complications soon after diagnosis. However, limited data exist about diabetes-specific distress (DD) and how diabetes teams can better support adolescents with T2D. We aimed to assess DD and other aspects of emotional/mental health among adolescents with T2D and qualitatively explore their lived experience and support needs.

**Methods:** This study used a cross-sectional mixed methods survey of adolescents with T2D, recruited via two tertiary diabetes clinics. Study outcomes included the Diabetes Distress Scale (DDS), World Health Organization-Five Well-being Index (WHO-5), Patient Health Questionnaire-2 (PHQ-2) and two free-text questions concerning what they wished their health professionals understood about living with T2D and diabetes support. Descriptive statistics and inductive thematic analysis were applied.

**Results:** Forty adolescents with T2D (22 females, predominantly from non-Indigenous background) completed all questionnaires. Nineteen were taking metformin, 18 were taking metformin plus injectables, and 3 were on lifestyle management. They had mean ± standard deviation (SD) age of 15.7 ± 2.1 years, median (interquartile range [IQR]) diabetes duration of 1.8 (0.8–2.6) years and median (IQR) glycated haemoglobin (HbA1c) of 6.9 (6.0–9.5)% (52 [42–80] mmol/mol). Twenty-one (53%) adolescents had moderate-to-severe DD, 16 (40%) had suboptimal emotional well-being, and 23 (58%) had depressive symptoms; 15 (38%) had both DD and depressive symptoms, while 11 (28%) had neither. Four themes described what adolescents wished their health professionals understood about living with diabetes: diabetes stigma, diabetes management burden, diabetes is challenging for young people and impact on mental health. Five themes described the support adolescents desired from their diabetes teams: show empathy and assist with motivation; mental health support; more frequent and convenient appointments; access to, and choice of, medications and management tools; and discussions about the future.

**Conclusions:** Most adolescents with T2D experience significant DD, impaired general emotional well-being and/or depressive symptoms. They also have considerable unmet support needs relevant to optimising their well-being and diabetes self-management.

## 1. Introduction

Worldwide, type 2 diabetes (T2D) is becoming increasingly prevalent among children and adolescents [1]. There is also increasing evidence that the onset of T2D at an earlier age is associated with a more aggressive disease progression [2] and earlier and more significant diabetes complications [3]. Psychological comorbidities are also highly prevalent amongst adolescents with T2D, with diabetes-specific distress (DD), the 'range of negative emotional responses (e.g. worry, fear, frustration, guilt, sadness, anger and overwhelm) to aspects of living with and managing diabetes, balanced against an appraisal of available coping resources' [4], being the least evaluated [5]. The two main scales validated to assess DD in adults include the Diabetes Distress Scale (DDS), which is mostly used in T2D and evaluates four domains, emotional burden, regimen-related distress, interpersonal distress and physician-related distress, and the Problem Areas in Diabetes (PAID) scale, which is mostly used for people with type 1 diabetes (T1D) [6]. The PAID-T, a modified version of the PAID, has been only validated for use in adolescents with T1D [7]. Neither version of the PAID evaluates physician-related distress, which is particularly relevant to T2D due to the associated stigma and adolescents' desire for reduced judgement and increased understanding from healthcare workers [8, 9].

Adolescents with T1D experience clinically significant DD, with an estimated prevalence of 33% using a variety of measures, including PAID, PAID-T and DDS [10]. Studies of DD in T2D are generally limited to older adult populations, reporting an overall prevalence of 36% [11]. A study of younger adults with T2D reported that over 90% had significant DD, and 57% had very severe DD [12]. The TODAY2 study found that 24% of young adults with T2D had DD [13]. DD holds both cross-sectional and time-varying associations with glycaemic control [14], and adults with significant DD are less likely to engage with patient education and self-management interventions [15, 16].

Only two studies have evaluated DD using the PAID-T [17, 18], and there is even less data on how paediatric diabetes teams can better support emotional well-being in adolescents with T2D. This notable gap must be addressed as recognition and management of DD by the diabetes care team are important [19]. Therefore, we aimed to evaluate DD levels using the DDS in adolescents with T2D and qualitatively explore their experiences of T2D to identify healthcare needs. We hypothesised that adolescents with T2D (1) experience DD and (2) have unmet needs in relation to diabetes care.

## 2. Methods

### 2.1. Study Design

We employed a cross-sectional mixed methods design, where adolescents responded to three validated psychometric measures and two open-ended questions. The Women's and Children's Human Research Ethics Committee (reference number 2021/HRE00376) approved the study. This ethics approval was accepted by the Child and Adolescent Health Service Human Research Ethics Committee in Perth, Western Australia (reference number: RGS0000005328); however, they did not approve the recruitment of Indigenous adolescents from their clinics as this would require a special separate ethics application. This project has been carried out in accordance with the Code of Ethics of the World Medical Association (Declaration of Helsinki).

All adolescents who completed the questionnaires provided informed written consent and/or assent, and all parents/guardians provided informed written consent. Adolescents in Western Australia were provided a link to the online survey at the outpatient clinic, and adolescents reviewed by telehealth were sent a link to the online survey. Online responses were collected using Research Electronic Data Capture (REDCap) (version 9.5.1, Vanderbilt University, Nashville, TN) software.

### 2.2. Subjects

Inclusion criteria included adolescents aged between 10 and 19 diagnosed with T2D for at least 1 month by a paediatric endocrinologist using the Australasian Paediatric Endocrine Group guidelines [20]. The exclusion criteria included diagnosis of T1D or other forms of diabetes (including monogenic diabetes), inability to speak English and having other communication difficulties or cognitive impairment.

Adolescents were consecutively recruited from the outpatient paediatric diabetes clinics at two Australian tertiary hospitals: the Women's and Children's Hospital, South Australia (between 2 March 2022 and 19 April 2023) and the Perth Children's Hospital, Western Australia (between 19 September 2022 and 20 April 2023).

### 2.3. Outcome Measures

The survey comprised three psychometric questionnaires and two open-ended answer questions. DD levels were measured using the DDS, a 17-item scale assessing four domains (regimen-related distress, physician-related distress, interpersonal distress and emotional burden) [6]. This study uses the DDS of Polonsky et al. [6], and the method description partly reproduces their wording. Adolescents indicated on a six-point Likert scale (1 = not a problem to 6 = a very serious problem), the degree to which each item distressed or bothered them during the past month; a higher score indicates a higher level of DD. A mean item score of 2–3 indicates a mild-to-moderate level of DD, and an overall score above 3 indicates severe DD [21].

Emotional well-being was assessed using the World Health Organization-Five Well-Being Index (WHO-5), a brief 5-item questionnaire that assesses general emotional well-being. Adolescents indicated, using a six-point Likert scale (0 = at no time to 5 = all of the time), how often in the past 2 weeks they experienced each statement; higher scores indicate greater well-being. A raw score ranging from 0 to 25 is multiplied by 4 to calculate a percentage score between 0 and 100, with a percentage score <50 indicating suboptimal emotional well-being. The WHO-5 is suitable for use in children aged 9 and above [22].

Risk of depression was measured using the two-item Patient Health Questionnaire-2 (PHQ-2), a screening tool that assesses the frequency of depressed mood and anhedonia over the preceding 2 weeks. Adolescents indicated, using a four-point Likert scale (not at all to every day), how often over the previous 2 weeks they had been bothered by each of the two problems described; higher scores indicate a greater risk of depression. Total scores can range from 0 to 6. A cut-off score of 2 or greater has the highest sensitivity and specificity for detecting depression in adolescents [23].

Adolescents' healthcare needs and experiences living with T2D were evaluated using two open-ended questions as follows: ‘What do you wish your health professionals understood about living with diabetes?' and ‘What can health professionals do to better support your overall wellbeing?' [24].

Adolescents and their families were advised that their healthcare practitioners would not see their questionnaire responses to minimise bias in the physician-related distress section of the DDS and responses to the open-ended questions. Adolescents were also asked to complete the questionnaires by themselves to minimise bias from parental involvement.

Demographic data were collected through adolescents self-reporting their age, gender, postcode, ethnicity, main language spoken at home, parental educational level and household structure. Additionally, diabetes self-management behaviour was measured with a short questionnaire evaluating the frequency of missed medications. Clinical data were obtained from adolescents' medical records from the closest visit to completion of the questionnaires. Clinical data included the date of diabetes diagnosis, glycated haemoglobin (HbA1c), medications for diabetes, the presence of comorbidities and complications of T2D, blood pressure, weight, height and body mass index (BMI) (kg/m^2^). Overweight and obesity were classified as having a BMI percentile between 85 and 95 and over 95, respectively [25]. BMI percentiles and *Z*-scores were calculated using software from the Children's Nutrition Research Center [26] based on appropriate Center for Disease Control paediatric growth charts [27].

### 2.4. Statistical Analysis

As the prevalence of DD in Australian adolescents with T2D was unknown, the sample size calculation was based on the prevalence (30%) of DD measured by PAID-T in mostly Hispanic adolescents [17]. To achieve a 95% confidence interval (CI) with a margin of error of 15% (actual prevalence could range between 15% and 45%), a minimum sample size of 36 was required.

Quantitative data were analysed using IBM SPSS Statistics (version 23.0, SPSS Inc., Chicago, IL) software. Continuous data are presented as mean ± standard deviation (SD) or median [interquartile range (IQR)] as appropriate, and categorical data are presented as counts and proportions.

Qualitative data, in the form of free-text responses, were screened for responses that could not be interpreted, including one-word answers. Such responses were excluded from the qualitative analysis. Following data familiarisation, a coding framework was developed in conjunction with Jane Speight and Melissa Oxlad, and coded responses were analysed inductively to generate key themes. The research team reviewed these themes to ensure they accurately represented the research data. All researchers agreed on the final themes.

## 3. Results

### 3.1. Participants

Participant eligibility screening and recruitment were undertaken and reported in accordance with the STROBE statement. Sixty-eight adolescents diagnosed with T2D for over 1 month who attended outpatient appointments at the Women's and Children's Hospital or the Perth Children's Hospital during the recruitment period were approached about the study.

Seventeen potential participants were ineligible due to significant cognitive impairment that prevented informed consent or comprehension of the study questionnaires (underlying pathologies including intellectual disability, autism, schizophrenia, chromosomal disorders and foetal alcohol syndrome). A further three adolescents were excluded due to either communication disorders or a language barrier that prevented completion of the written English questionnaires. Of the 48 eligible adolescents approached for the study, 40 (83%) completed all the questionnaires and were included in the analysis. Eight did not complete the online survey sent via REDcap after the appointment.

Adolescent's characteristics are summarised in Table 1. Most adolescents were overweight or obese (90%), from non-Indigenous backgrounds (92.5%) and resided in metropolitan areas (80%).

### 3.2. Diabetes Distress and Emotional Well-being

The median (IQR) score on the DDS was 2.0 (1.3–2.8), which was the cut-off for clinically significant DD. The median (IQR) highest domain was regimen-related distress (2.5 [1.6–3.6]), followed by emotional burden (2.2 [1.4–3.6]). Median (IQR) scores on the physician-related distress (1 [1–1.3]) and interpersonal distress (1 [1–1.7]) domains were the lowest possible scores. The proportion (95% CI) of adolescents with some DD was 52.5% (36.1–68.5). The median (IQR) WHO-5 score was 64 (40–72%), which is within the normal range. The proportion (95% CI) of adolescents with suboptimal emotional well-being was 40% (24.9–56.7). The median (IQR) PHQ-2 score was 2.0 (0–3), which is at the cut-off for increased risk of major depressive disorder. The proportion (95% CI) of adolescents with depressive symptoms was 57.5% (38.5–70.7).

According to patient interviews and medical records, five adolescents (12.5%) had previously been diagnosed with major depressive disorder; three of these adolescents were being treated with antidepressant medication. Using the PHQ-2, 23 adolescents (57.5%; including the five participants previously diagnosed with major depressive disorder) reported depressive symptoms above the cut-off indicative of likely major depressive disorder. For this group of 23 participants, their general practitioner was informed, and they were referred to a psychologist for further management.

**Figure 1:**
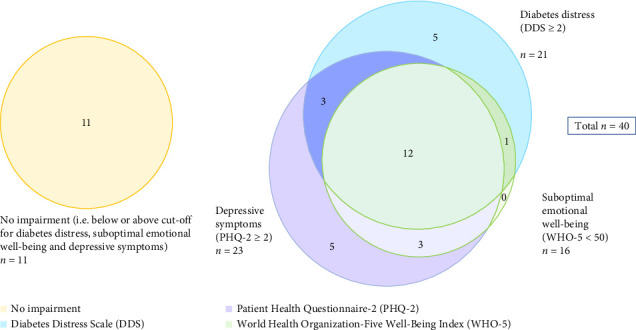
Cumulative emotional health burden (diabetes distress, emotional well-being and depressive symptoms). *Note*. The area of each circle is representative of the total sample size in that group.

There was a moderate degree of overlap between the results of the questionnaires which represents the cumulative emotional health burden as shown in Figure 1. Eleven adolescents had no DD, suboptimal emotional well-being or depressive symptoms, while the remaining 29 had at least one of these features. The cumulative emotional health burden including DD and depressive symptoms is shown in Table 2.

### 3.3. Qualitative Data Summarising Adolescents' Experiences Living With T2D

In response to the question, ‘What do you wish your healthcare professionals understood about your experience living with T2D?', four themes emerged: diabetes stigma, diabetes management burden, diabetes is challenging for young people and impact on mental health (Table 3).

### 3.4. Qualitative Data Summarising Adolescents' Desired Support From Their Healthcare Team

In response to the question, ‘Is there anything that your doctors or healthcare professionals could do to better support you?', five themes were generated: show empathy and assist with motivation; mental health support; more frequent and convenient appointments; access to, and choice of, medications and management tools; and discussions about the future and what to expect (Table 4).

## 4. Discussion

We aimed to evaluate DD levels in adolescents with T2D and qualitatively explore their experiences of T2D to identify healthcare needs. Furthermore, we hypothesised that adolescents with T2D (1) experience DD and (2) have unmet needs regarding their diabetes care. Our findings supported our hypotheses, with a large proportion of adolescents with T2D experiencing DD and many describing unmet diabetes care needs, especially in relation to mental health support.

The prevalence of DD in our study was higher than previously reported in an American study including African American and Hispanic adolescents [17, 18]. This difference may be due to our use of DDS, which is specific to T2D (unlike the PAID or PAID-T used in past studies) and, therefore, is more likely to identify DD in adolescents with T2D. Additionally, adolescents in our study came from diverse multicultural backgrounds (Asian, Caucasian, Middle Eastern, African and Indigenous), highlighting that DD may be higher in these groups.

As our study is the first to assess DD in adolescents with T2D using DDS, we were unable to directly compare our findings to past research with the same population. Luo et al. [28] used DDS in Chinese adolescents with T1D, finding the most elevated domain to be physician-related distress, followed by interpersonal distress, regimen-related distress and emotional burden, respectively. These findings contrast ours, where the most elevated domain was regimen-related distress, followed by emotional burden, physician-related distress and interpersonal distress. These differences may be due to unique experiences associated with diabetes type (i.e. T1D vs. T2D) and/or contextual/cultural and healthcare system differences (i.e. China vs. Australia).

Our results also demonstrated a moderate degree of overlap between DD, suboptimal emotional well-being and depressive symptoms. These results were expected based on current literature suggesting that DD, poor emotional well-being and depressive symptoms are clinically distinct entities that can often coexist in high-risk individuals [5]. While only 5 of our 40 adolescents had previously been diagnosed with major depressive disorder, 23 (including the 5 previously diagnosed with major depressive disorder) reported depressive symptoms above the PHQ-2 cut-off indicative of likely major depressive disorder. As these results warranted further evaluation, these adolescents' general practitioner was informed, and they were referred to a psychologist for further management. Our findings reiterate the importance of the guidelines and position statement recommendations that routine screening for mental health using validated measures should be integrated into clinical encounters with people with diabetes (i.e. initial consultation and regular appointments, particularly when a patient may experience changes in disease, treatment or life circumstances) [20, 29–31]. Consistent with our quantitative findings, our adolescents' qualitative responses indicate a desire for increased mental health support during diabetes clinic visits. Thus, our findings reinforce the need for widespread implementation of current guidelines that recommend having psychosocial care providers embedded in diabetes care settings [30].

Stigma, social isolation and the mental health impact of T2D noted in our adolescents' free-text responses reflected findings in the MILES-2 study of 1316 adults with T2D, which used the same qualitative question concerning what people with diabetes wished their healthcare professionals understood about their experience living with diabetes [24]

The themes in our study of stigma, social isolation and the burden of managing T2D were comparable to the findings of two focus-group studies reporting the experiences of Indigenous Canadian [9] and American (predominantly African American) adolescents with T2D [32]. Indigenous Canadian adolescents described ‘weight' or burden of diabetes and experienced stigma and shame related to their diagnosis, which was a barrier to their diabetes self-management. Furthermore, these adolescents described the feeling that others, including healthcare professionals, blamed them for their T2D and wanted healthcare professionals to be more understanding [9]. In American adolescents with T2D, social isolation was reduced, and emotional support increased when adolescents gained a sense of belonging by forming connections with other people with diabetes. Such connections provided valuable emotional support, with adolescents feeling understood by peers also managing diabetes [32].

In Australia, researchers, using semistructured interviews, explored the experiences of 16 Indigenous (Aboriginal and Torres Strait Islander) young people (aged 11–20) with T2D [33]. Weaver et al. [33] reported two themes, which were not present in our results: a ‘normalisation' in response to T2D diagnosis (adult diabetes is common among their communities) and ‘suboptimal levels of understanding of T2D'. This variation in themes may be partly due to differing research methods; Weaver et al. [33] used semistructured interviews that covered multiple topics and enabled researchers to ask further follow-up prompts as needed, whereas our qualitative data comprised two open-ended questions for which adolescents could provide free-text responses. This approach meant we could not follow-up on adolescents' responses for further information. Additionally, theme variation may have occurred due to sociocultural factors and differing needs and experiences of Indigenous and non-Indigenous adolescents with T2D, as most of our sample was predominantly from non-Indigenous backgrounds. It would have been interesting to have more Indigenous adolescents in our study to be able to compare results between different Indigenous cohorts (Canadian and Australian) and between Indigenous and non-Indigenous groups (culturally diverse groups), as both groups have strong family histories of T2D.

Strengths of the study include the following: this is the first to evaluate DD (using the DDS) and emotional well-being in adolescents with T2D and the first Australian study to qualitatively explore how healthcare teams can better support adolescents with T2D from predominantly non-Indigenous backgrounds. Additionally, our inclusion of free-text questions allowed adolescents to share, in their own words, their experiences living with T2D and perspectives about how their healthcare team could better support them. The main limitation of our study was the relatively small sample size of 40 adolescents; however, our sample was recruited from two paediatric centres and comparable in age (15.2 years), age at diagnosis (13.9 years) and gender (55%) to the reported by the Australasian Diabetes Data Network (ADDN) [34]. Our study had a higher proportion of adolescents from culturally diverse backgrounds than the general Australian adolescent population but is consistent with the higher prevalence rate of T2D in high-risk ethnic groups [35]. Additionally, our sample comprised of fewer Indigenous adolescents than the ADDN data (7.5% vs. 23%). This difference might be explained by the additional requirement of gaining site-specific ethical approval to include Indigenous adolescents at one of the centres. While overall our findings appear largely generalisable, the extent of generalisability is impacted by the lack of recruitment from rural clinics and the lower proportion of Indigenous adolescents.

Another weakness of the study is that the DDS has not been validated in adolescents with T2D; however, no measure has been designed for this purpose, and the DDS has been validated in adults with T2D. Additionally, adolescents in our study understood and found the questions relevant to living with T2D. Given the paucity of research examining DD in adolescents with T2D, further research to gain greater certainty about the levels of DD using a T2D-appropriate measure would be beneficial. Additionally, researchers should seek a greater understanding of DD among Indigenous adolescents with T2D, adolescents managed by general practitioners in nonmetropolitan communities and internationally due to variations in healthcare systems and approaches. Finally, conducting interviews and/or focus groups with adolescents and their families would enable the development of a more in-depth understanding of the impacts of managing this health condition and individuals' healthcare needs.

## 5. Conclusion

This is the first study globally to evaluate DD (with DDS) and emotional well-being and to qualitatively explore how healthcare teams can better support adolescents with T2D. Our results indicate that DD, suboptimal emotional well-being and depressive symptoms are common among adolescents with T2D. Additionally, many adolescents desire greater support from their healthcare teams, particularly concerning mental health. Our findings offer significant insights to inform further research and clinical practice. Our findings suggest that adolescents with T2D and their families would benefit from having psychosocial care providers, such as psychologists, embedded in diabetes care settings to increase access to mental health support from professionals who understand the impacts of diabetes.

## Figures and Tables

**Table 1:** Adolescents' characteristics.

Variables	Adolescents^a^ (*n* = 40)
Age (years) (mean ± SD)	15.7 ± 2.1
Gender	
Male	18 (45.0)
Female	22 (55.0)
Ethnicity	
Asian	14 (35.0)
Caucasian	11 (27.5)
Middle Eastern	4 (10.0)
African	4 (10.0)
Indigenous Australian	4 (10.0)
Polynesian	3 (7.5)
Main language spoken at home	
English	24 (60.0)
Language other than English	16 (40.0)
Residence	
Metropolitan	32 (80.0)
Rural	8 (20.0)
Household structure	
Dual-parent household	29 (72.5)
Single-parent household	8 (20.0)
Split household	1 (2.5)
Kinship arrangement	2 (5.0)
Parental tertiary education	
Neither parent	18 (45.0)
One parent	9 (22.5)
Both parents	11 (27.5)
Unknown	2 (5.0)
Type 2 diabetes	
Duration (years) (median [IQR])	1.8 (0.8–2.6)
Age at diagnosis (years) (mean ± SD)	13.7 ± 2.2
Diabetes management regimen (*n*)	
Lifestyle alone	3 (7.5)
Metformin	19 (47.5)
Metformin + insulin	10 (25.0)
Metformin + insulin + glucagon-like peptide 1 receptor agonist	6 (15.0)
Metformin + insulin + glucagon-like peptide 1 receptor agonist + sodium glucose cotransporter 2 inhibitor	1 (2.5)
Metformin + sodium glucose cotransporter 2 inhibitor	1 (2.5)
Missed medications	
Never	12 (30.0)
Sometimes	14 (35.0)
Often	2 (5.0)
Most of the time	5 (12.5)
All of the time	4 (10)
Not applicable (lifestyle alone)	3 (7.5)
Complications of diabetes	
Microalbuminuria	11 (27.5)
Retinopathy	1 (2.5)
No complications	28 (70.0)
Family history T2D	
Parent alone	15 (37.5)
Other family member alone	5 (12.5)
Parent + other family member	10 (25.0)
None	10 (25.0)
HbA1c (%) median (IQR)	6.9 (6.0–9.5)
HbA1c (mmol/mol) median (IQR)	52 (42–80)
Body mass index (BMI) (median [IQR])	
Raw score (kg/m^2^)	32.2 (27.3–39.7)
*Z*-score	2.2 (1.4–2.6)
BMI by category	
Underweight	0 (0.0)
Normal weight	4 (10.0)
Overweight	7 (17.5)
Obese	29 (72.5)

Abbreviations: HbA1c, glycated haemoglobin; IQR, interquartile range; SD, standard deviation; T2D, type 2 diabetes.

^a^Data are reported as *n* (%) unless otherwise specified.

**Table 2:** Cumulative emotional health burden (diabetes distress and depressive symptoms).

		Diabetes distress (DDS ≥ 2)		
		Yes	No	Total
Depressive symptoms (PHQ-2 ≥ 2)	Yes	15	8	23
	No	6	11	17
	Total	21	19	40

*Note*: Data are *n*.

Abbreviations: DDS, Diabetes Distress Scale; PHQ-2, Patient Health Questionnaire-2.

**Table 3:** What adolescents wish their healthcare professionals understood about them living with T2D.

Theme	Examples of adolescents' responses
Diabetes stigma	‘I worry sometimes they may judge me or think that it's my fault that I have diabetes…' ‘It[s] kind of hard to inject insulin in front of everyone'

Diabetes management burden	‘How hard it is to really manage taking the insulin. … the pain of injecting insulin 3 times per day' ‘Restrictions in diet and fear of high glucose when testing' ‘That exercising and sticking to a plan is harder than they may think'

Diabetes is challenging for young people	
Subtheme: It is hard	‘I wish they knew that it's definitely hard living with diabetes as a teenager…' ‘That it can be hard at times for everyone and to be patient with whoever they are seeing because changing your lifestyle all of a sudden can be hard and frustrating due to diabetes'
Subtheme: Responsibility	‘It is difficult living with diabetes as it impacts the way that I enjoy life' ‘Being a kid it can be too much responsibility—especially taking tablets'
Subtheme: Social isolation	‘I'm young and none of my friends have it' ‘The specific effects that diabetes has as a teenager … Social Isolation'
Subtheme: Balancing life-stage commitments with managing diabetes	‘School and work is constantly taking over my time and I don't have time to plan my meals or keep up with my medications' ‘How hard it is to really manage taking the insulin. Balancing work, uni and diabetes all at once … the reality of going back to everything normally after diabetes is diagnosed'

Impact on mental health	‘My mental health gets worse with diabetes' ‘…Taking medication and exercise, and a healthy diet plan … at the end of the day, it doesn't matter when all mental illness will allow us to do is lie in bed and waste away' ‘Increased depression risk and lack of motivation'

Abbreviation: T2D, type 2 diabetes.

**Table 4:** Support desired by adolescents with T2D from their healthcare team.

Theme	Examples of adolescents' responses
Show empathy and assist with motivation	‘My doctors are supportive of my wellbeing' ‘Checking in with wellbeing' ‘Try to understand more about living a life with diabetes' ‘I think a bit more support on how to stay motivated' ‘They could help me with my motivation'

Mental health support (including integration of mental health professionals as part of diabetes team)	‘…Have more support groups available, have more open discussions about the impact of diabetes on mental health' ‘…giving options on how to deal with the mental wellbeing of living with diabetes' ‘Provide mental health care as part of the diabetic team, as it would help us to manage our mental health in order to keep up with the demands of the condition, especially, during the first few months of diagnosis' ‘It might be nice to have a psychologist to talk to who understands what it can be like'

More frequent and convenient appointments	‘Having more appointments would help me manage my diabetes more often and would keep me in check' ‘Have weekly catch-ups with patients…' ‘Regular check-up' ‘Closer to my appointments. Not having to travel a long way all the time' ‘Come to the country'

Access to, and choice of, medications and management tools	‘Make medication free' ‘….More devices for Type 2 diabetics for blood sugars' ‘Different medications, having access to a glucose monitor on my arm…'

Discussions about the future and what to expect	‘Elaborate and give a better brief on the future with diabetes, rather than just the now!' ‘Support and talk about how our bodies will change over time'

## Data Availability

De-identifiable data are available upon reasonable request as per ethics requirements.

## Supporting Information

Supporting Information STROBE—L checklist.
